# Macrophages produce PGE2 to promote hepatic stellate cell autophagy and liver fibrosis

**DOI:** 10.1080/27694127.2022.2119513

**Published:** 2022-09-04

**Authors:** Liuluan Zhu, Yanxi Zhou, Rui Li, Shuwei Deng

**Affiliations:** a Beijing Key Laboratory of Emerging Infectious Diseases, Institute of Infectious Diseases, Beijing Ditan Hospital, Capital Medical University, Beijing 100015, China; b Beijing Institute of Infectious Diseases, Beijing 100015, China; c National Center of Infectious Diseases, Beijing Ditan Hospital, Capital Medical University, Beijing 100015, China

**Keywords:** Autophagy, E2, E7046, liver fibrosis, macrophage polarization, nonalcoholic fatty liver disease, prostaglandin EP4

## Abstract

The pathogenesis of liver fibrosis in nonalcoholic fatty liver disease (NAFLD) remains unclear and the effective treatments have not been explored yet. The activation of hepatic stellate cells (HSCs) is the most critical factor in the progression of liver fibrosis. Macroautophagy/autophagy has recently been identified as a new mechanism to regulate HSC activation. In a recent study, we found that type 2 (M2) macrophages promote HSC autophagy by secreting prostaglandin E2 (PGE2) to bind its receptor PTGER4/EP4 on HSCs, consequently activating the MAPK/ERK pathway to promote autophagy and activation of HSCs. A specific PGE2-PTGER4 antagonist, E7046, significantly inhibits HSC autophagy and improves liver fibrosis and histopathology in NAFLD mice. Our findings provide novel mechanistic insights into liver fibrosis and suggest E7046 as a promising therapy to prevent NASH progression.

NAFLD has a global prevalence of 25% and is a leading cause of cirrhosis. Once cirrhosis is advanced, the risk of developing hepatocellular carcinoma is significantly increased. Cirrhosis is an important factor for liver-related morbidity and mortality in patients with NAFLD, which is the result of dynamic progression of liver fibrosis. Thus, prevention of liver fibrosis is the key to delaying the progression of cirrhosis and hepatocellular carcinoma. Unfortunately, there is currently no approved treatment for liver fibrosis. These facts highlight the need for a deeper comprehension of the pathogenesis of liver fibrosis and the translation of this knowledge into new therapeutic approaches.

The core pathogenesis of liver fibrosis is the activation of hepatic stellate cells (HSCs). Under various stimulations, quiescent HSCs are activated and transdifferentiated into myofibroblasts and generate a large amount of extracellular matrix, which is the characteristic change of liver fibrosis. Recently, autophagy has been identified as a new mechanism to regulate HSC activation. Therefore, further revealing signals triggering the autophagy of HSCs will provide new insights into the intervention of NAFLD-related liver fibrosis. In a recent paper [1], we adopted a widely used NAFLD model by using a methionine- and choline-deficient diet for several weeks and determined that the polarization of hepatic macrophages shifts from M1 to M2 during the progression of NAFLD to NASH in mice, accompanied by autophagy and activation of HSCs. This phenomenon was also observed in human patients with NAFLD and NASH.

Macrophages are important immune cells in innate immunity and have remarkable heterogeneity and polarization. Under pathological conditions, in addition to the resident macrophages, other macrophages are also recruited to the diseased tissues, and polarize to various phenotypes (mainly M1 and M2) under the stimulation of various factors in the microenvironment, thus playing different roles and functions. In this study, we first identified the regulatory role of macrophages in HSC autophagy. By constructing three co-culture systems of polarized macrophages and HSCs—mixed culture, transwell culture, and M2 macrophage-conditioned medium culture—we demonstrated that M2 macrophages promote autophagy and activation of HSCs independent of cell‑cell contact. This finding implies that the regulation depends on a secretory pathway. Subsequently, we treated LX-2 cells with recombinant proteins of M2 macrophage-secreted cytokines and measured autophagy and activation. We found that among the cytokines, only PGE2, rather than TGFB/TGF-β, the profibrogenic core factor, has the ability to promote HSC autophagy.

PTGER4 (prostaglandin E receptor 4 (subtype EP4)) is a primary receptor for PGE2. As expected, we found that the expression of PTGER4 is increased in LX-2 cells after supplementing with M2 macrophage-conditioned medium. Then we used a specific antagonist, E7046, to block the binding of PGE2 to PTGER4. E7046 shows a significant inhibition of the upregulation of ACTA2/α-SMA induced by M2 macrophage-conditioned medium or recombinant PGE2. E7046 also restrains PGE2-triggered conversion from LC3B-I to LC3B-II and the formation of autophagosomes. Consistently, the silencing of PTGER4 in LX-2 cells with shRNA reduces the expression of autophagy genes in LX-2 cells induced by PGE2. These data collectively support the idea that M2 macrophages promote autophagy and activation of HSCs through the PGE2-PTGER4 pathway. Further mechanistic analysis reveals that the PGE2-PTGER4 signal regulates HSC autophagy by activating the MAPK/ERK pathway, independent of the classical autophagic AKT-MTOR pathway. Moreover, oral treatment with E7046 reduces collagen fiber deposition and relieves liver damage in mice with NASH. Collectively, using a variety of *in vitro* and *in vivo* experimental methods, we found that liver macrophages polarize toward M2 and promote HSC autophagy by secreting PGE2 and binding its receptor PTGER4 on the surface of HSCs during the progression of NAFLD. These findings reveal a novel mechanism of liver fibrosis independent of the classical TGFB pathway, and may explain part of the reason for the non-response to TGFB therapy. E7046 is a highly selective, small-molecule antagonist PTGER4, displaying a robust anti-tumor activity by diminishing myeloid immunosuppression *in vivo* and *in vitro*. Furthermore, two clinical trials (NCT02540291 and NCT03152370) have been launched to treat patients with cancers; these studies provide valuable insights into the safety of E7046 in human patients for treatment of liver fibrosis.

In addition to the regulation of M2 macrophages on HSC activation, we also demonstrated that the activated HSCs promote M2 macrophage polarization in reverse (Figure 1). The cross-talk between HSC and macrophage, and many other hepatic cells, involves the hepatic microenvironment that is complicated and essential at various stages of NAFLD and fibrogenesis. Our study and recent advances suggest that the hepatic microenvironment represents a key aspect leading to the progression toward cirrhosis, whereas many facets of this interaction have yet to be thoroughly explored. Future studies will be necessary to explore the mechanisms of macrophage polarization in different stages and microenvironments of NAFLD. A comprehensive understanding of the liver metabolism-immune microenvironment is necessary to fully understand the pathogenesis of NAFLD and will also provide a basis for exploring therapeutic targets.

**Figure 1.**
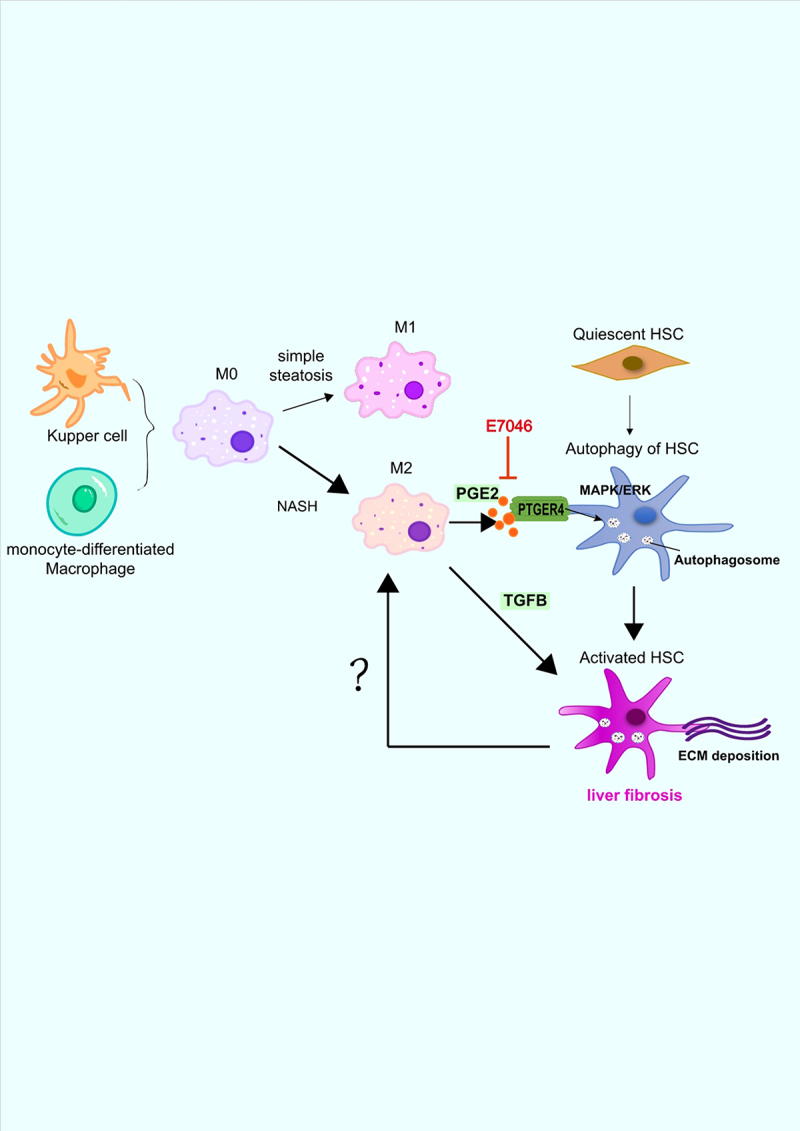
Schematic of the regulation between macrophages and HSCs in the progression of NAFLD. At the stage of simple steatosis, liver macrophages polarize toward type 1 (M1) and cause inflammation. With the progression into NASH, macrophages polarize shifting from M1 to M2, and secret cytokines including PGE2 and TGFB to resolve the inflammation and repair the injured liver tissue. PGE2 binds to its receptor PTGER4 on the surface of HSCs to activate the MAPK/ERK signaling pathway promoting autophagy and activation, which can be blocked by the specific antagonist E7046. The activated HSCs transdifferentiate into myofibroblasts to generate a large amount of extracellular matrix (ECM) and expedite M2 macrophage polarization. The positive feedback between macrophages and HSCs accelerates the progression of liver fibrosis.
